# Concurrent Onset of Central Retinal Vein Occlusion and Inflammation of a Large Maxillary Odontogenic Cyst: Case Report and Analysis

**DOI:** 10.3390/reports7030055

**Published:** 2024-07-14

**Authors:** Vlatko Kopić, Andrijana Kopić, Mihael Mišir, Sanjin Petrović

**Affiliations:** 1 Department of Maxillofacial and Oral Surgery, University Hospital Centre Osijek, 31000 Osijek, Croatia; 2 Faculty of Medicine Osijek, Josip Juraj Strossmayer University of Osijek, 31000 Osijek, Croatia; 3 Department of Ophtalmology, University Hospital Centre Osijek, 31000 Osijek, Croatia; 4 Department of Neurology, University Hospital Centre Osijek, 31000 Osijek, Croatia

**Keywords:** retinal vein occlusion, central retinal vein occlusion, odontogenic cyst, maxillary cyst, vision loss, odontogenic infection

## Abstract

Central retinal vein occlusion typically manifests in older individuals with underlying systemic pathology, leading to a spectrum of symptoms ranging from blurred vision to complete vision loss. While odontogenic infections are recognized for causing complications affecting the eye and vision, their potential role as an etiological factor in cases of sudden vision impairment merits consideration. This article presents a case involving central retinal vein thrombosis, wherein resolution was achieved through a combination of ophthalmic therapy and the surgical removal of a concurrently existing large inflamed odontogenic cyst located in the ipsilateral hemimaxilla. This case underscores the importance of recognizing odontogenic factors in the assessment of sudden vision impairment and the efficacy of a multidisciplinary therapeutic approach.

## 1. Introduction

Retinal vein occlusion (RVO) predominantly afflicts elderly individuals, with a higher prevalence observed in those with comorbidities such as hypertension, glaucoma, arteriosclerosis, and diabetes [1,2]. Statistical evidence suggests that merely 10% to 15% of afflicted individuals fall below the age of 40 [3]. Ranking as the second most prevalent vascular retinal disorder following diabetic retinopathy, RVO stands as a frequent cause of vision impairment in the elderly [4]. If the occlusion occurs at the lamina cribrosa of the optic nerve, where the vein exits the eye, or proximal to it, the condition is called central retinal vein occlusion (CRVO). This occlusive event leads to a spectrum of symptoms ranging from blurred vision to complete vision loss. Additionally, a variant known as branch retinal vein occlusion (BRVO) is discernible, representing a more commonplace occurrence where occlusion arises at any branch of the central retinal vein, typically accompanied by milder symptomatology. Some RVO classifications include another, separate type of occlusion called hemi-RVO (HRVO), which involves the anterior portion of the trunk of the central retinal vein [5].

CRVO can be further subdivided into non-ischemic (perfused) and ischemic (non-perfused) types based on retinal perfusion. The pathogenesis remains elusive; however, it is postulated to adhere to Virchow’s triad principles for thrombogenesis and may be linked to pathological changes in the central retinal artery, given their shared adventitial sheath [6]. Clinical diagnosis of CRVO entails meticulous examination, revealing distinctive fundoscopic manifestations such as tortuosity and dilatation of all central retinal vein branches, dot/blot hemorrhages, and optic disc and macular edema. Confirmation is achieved through fluorescein angiography (FA) and optical coherence tomography (OCT). There are a variety of treatment options available, and their effectiveness depends on the extent of hypoxic damage. Timely and accurate diagnosis, coupled with patient compliance, is pivotal for optimal outcomes [7,8]. Treatment modalities encompass intravitreal anti-VEGF and corticosteroid treatment, laser photocoagulation, and, in the presence of complications, antiglaucoma therapy and surgical interventions [9].

## 2. Case Presentation

**Figure 1:**
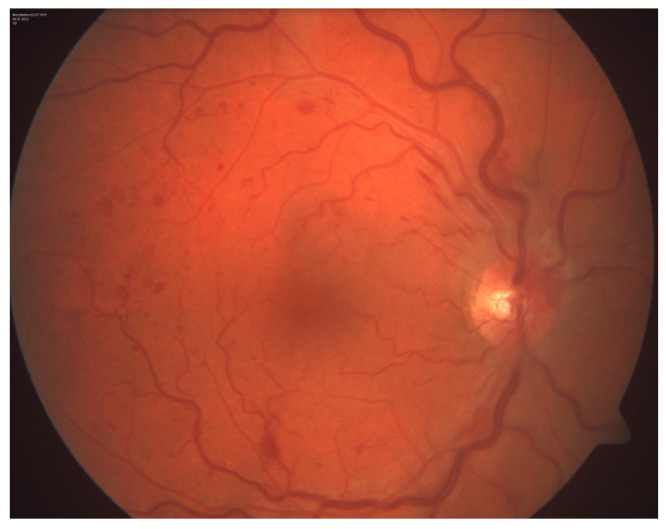
Photography of the ocular fundus of the right eye.

A 47-year-old previously healthy female sought evaluation at the Department of Ophthalmology, University Hospital Centre Osijek, presenting with complaints of blurred vision and flashes affecting her right eye exclusively. No accompanying symptoms were reported. The right eye’s best-corrected visual acuity (BCVA) measured 0.8 according to the Snellen chart. Fundoscopic examination revealed features indicative of CRVO, characterized by discrete edema without macular involvement and tortuosity with dilatation of all branches of the central retinal vein (Figure 1).

**Figure 2:**
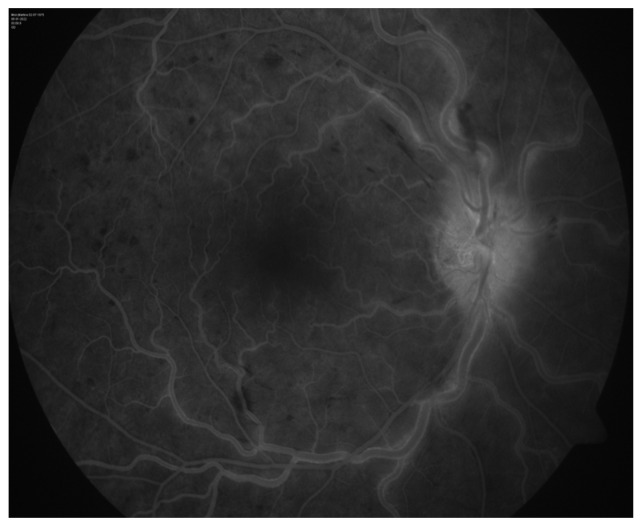
Fluorescein angiography of the right eye.

Clinical diagnosis was confirmed by FA, which showed the absence of ischemic zones (Figure 2).

**Figure 3:**
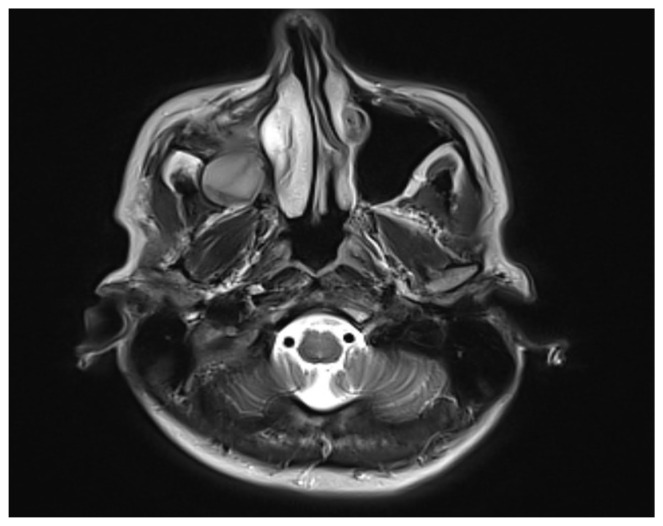
MRI scan of the head showing maxillary sinus cyst.

Laboratory results disclosed marked leukocytosis (37.9 × 10^9^/L) and a slightly elevated fibrinogen concentration (4.5 g/L). Daily oral intake of 100 mg acetylsalicylic acid along with vitamin C was recommended. As part of a comprehensive diagnostic evaluation, a head MRI revealed a well-defined oval zone within the right maxillary sinus with signal intensity indicative of a viscous fluid. No other significant pathological changes were observed (Figure 3).

Subsequently, the patient was referred to the Department of Oral and Maxillofacial Surgery. Thorough review of the patient’s history revealed a prior right maxillary sinus surgery ten years earlier for empyema.

**Figure 4:**
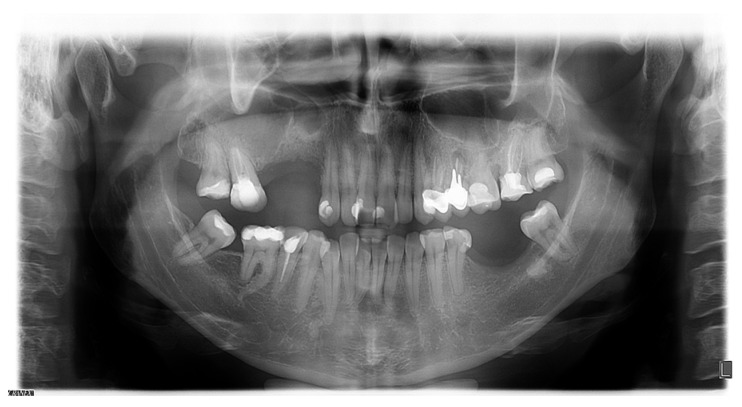
Orthopantomography.

Clinical examination identified a tender swelling in the upper right vestibule of the oral cavity. Orthopantomography showed the root of tooth 17, which protruded into the sharply limited cystic cavity and filled the right maxillary sinus. Orthopantomography also showed inadequate endodontic treatment of tooth 17 (Figure 4).

**Figure 5:**
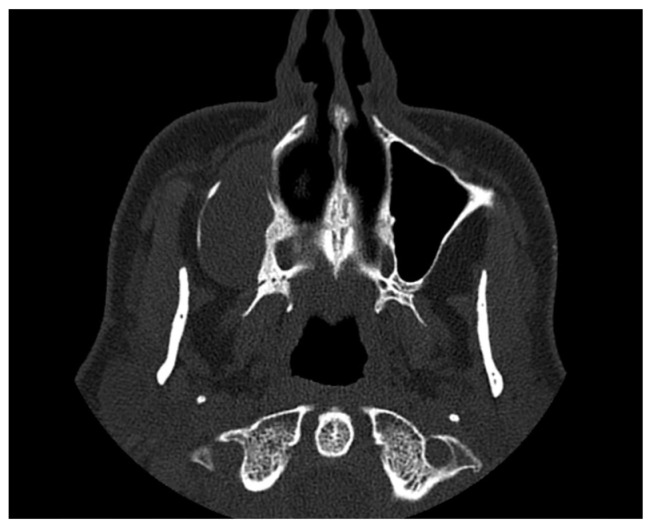
MSCT of the paranasal sinuses.

Subsequent multi-slice computed tomography (MSCT) of the paranasal sinuses demonstrated an oval radiolucency in the alveolar bone and alveolar recess of the right maxillary sinus, measuring 40 mm in diameter, with destruction of the surrounding lateral bone walls (Figure 5). Other than a cyst, a possible differential diagnosis was gigantocellular granuloma.

**Figure 6:**
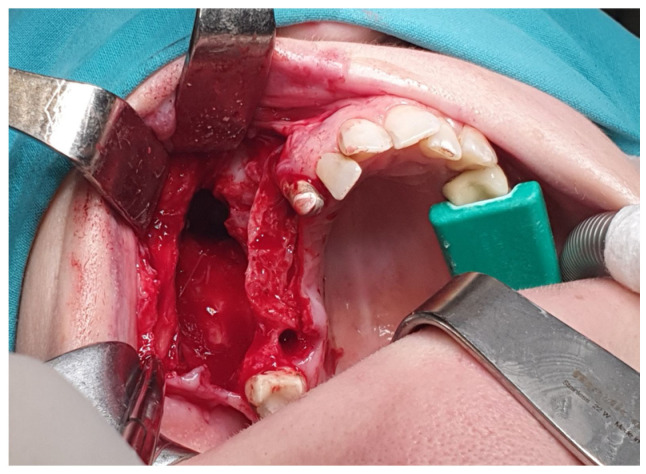
Surgical procedure of the cyst enucleation.

Needle aspiration and cytologic smear of the cystic content revealed greenish viscous fluid containing phagocytes, preserved and degenerated granulocytes, erythrocytes, and detritus—consistent with the liquid content of an inflamed cyst. Surgical intervention comprised cyst enucleation and Caldwell–Luc antrostomy (Figure 6).

Oral antiplatelet therapy was replaced by daily subcutaneous administration of 60 mg enoxaparin. Concurrent parenteral antimicrobial therapy with clindamycin and ceftriaxone was initiated. Histological analysis confirmed the inflammatory odontogenic origin of the enucleated cystic lesion, identified as a radicular cyst. The primary surgical procedure precipitated a postoperative complication in the form of oroantral communication, prompting a subsequent, second surgical intervention, which effectively resolved the defect.

**Figure 7:**
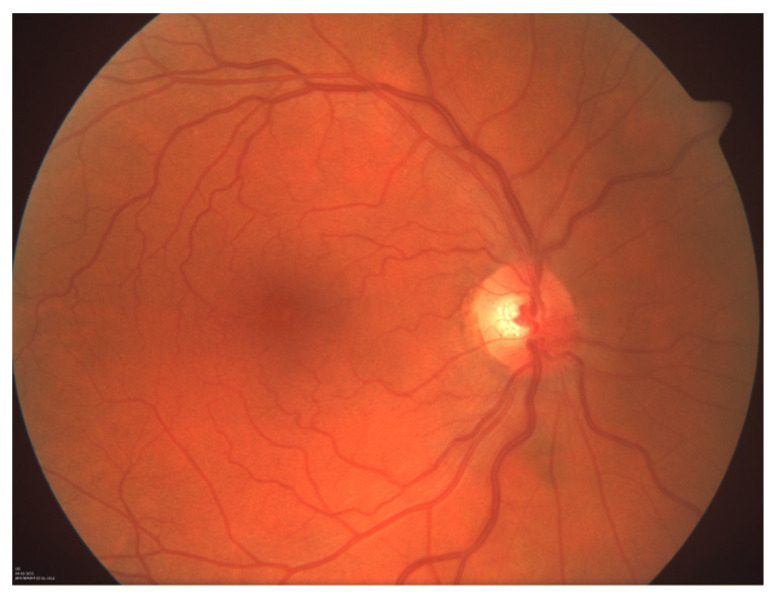
Photograph of the ocular fundus of the right eye showing recovery after the surgical treatment.

Intravitreal anti-VEGF injections, initially planned as the primary modality for ophthalmic CRVO treatment, were deferred due to purulent inflammation in the adjacent maxillary area and the associated risk of post-injection endophthalmitis. Instead, topical application of dexamethasone and bromfenac drops were recommended. Ophthalmic follow-ups conducted at two weeks, two months, and four months after surgical removal of the inflamed cystic lesion demonstrated progressive improvement in visual acuity and resolution of retinal damage (Figure 7).

The follow-up is now at nearly two years, and our patient shows no signs of relapse of the cyst or any visual disturbances.

## 3. Discussion

We present a case report detailing a relatively uncommon occurrence of CRVO concomitant with inflammation of a large odontogenic cyst within the ipsilateral hemimaxilla, the symptomatic resolution of which was largely achieved following surgical cyst removal.

Based on the extensive cross-sectional population-based survey conducted by Foreman and colleagues, several prevalent factors contribute to unilateral vision impairment or blindness. These include uncorrected refractive error, cataract, corneal pathology, amblyopia, trauma, and age-related macular degeneration. Notably, retinal vein occlusion (RVO) constitutes a relatively small proportion of cases among the aforementioned pathologies [10].

Ocular complications arising from odontogenic infections are infrequent, with approximately 40% attributed to intravascular propagation of infection [11]. The established understanding delineates that venous drainage originating from the midfacial region is directed through the ophthalmic veins. Contrary to frequent assertions of complete valvelessness, these veins exhibit some valvular structures. Notably, this anatomical configuration permits the bidirectional propagation of infection, encompassing both retrograde and anterograde pathways [12]. The pathophysiology of CRVO remains unclear, which is the reason for the lack of a single highly successful treatment. Current therapeutic modalities predominantly address complications rather than the underlying etiology. Presumed pathophysiological mechanisms encompass hypercoagulability, thrombus formation, and platelet dysfunction [13].

Our patient exhibited slightly elevated fibrinogen levels on initial laboratory evaluation, a parameter implicated as a potential risk factor for CRVO. Elevated fibrinogen is strongly associated with vascular diseases, augmenting blood viscosity, fostering fibrinogenesis, and intensifying platelet interactions [14,15]. Additionally, it serves as a marker for peripheral inflammation [16].

As part of the secondary prevention measures aimed at reducing the probability of a recurrent RVO episode, our patient was prescribed vitamin C. Intake of this molecule has been associated with a reduction in the caliber of retinal veins, resulting in an improved quality of retinal microcirculation [17,18]. Additionally, vitamin C is known to possess properties that inhibit the activation of inflammatory mediators such as cytokines and chemokines, which play a role in the pathogenesis of RVO [19].

Antithrombotic therapy plays a crucial role in enhancing visual acuity and mitigating the risk of recurrent RVO. In our case, an antiplatelet agent was selected, yielding no complications. However, emerging evidence suggests that the utilization of anticoagulants may offer superior efficacy and safety profiles [20].

Optimal management of odontogenic cysts remains contentious [21]. Common surgical interventions involve enucleation, marsupialization, and decompression. In cases involving large cysts impacting vital anatomical structures or necessitating devitalization of multiple teeth, conservative approaches such as decompression may prove beneficial [21]. Decompression has consistently demonstrated its utility and effectiveness as a primary treatment for jaw cysts. However, several drawbacks accompany this approach. These include the necessity for additional surgical interventions following the initial procedure, prolonged therapy duration, requisite patient commitment and compliance, multiple follow-up appointments, and the risk of lesion infection stemming from its communication with the oral cavity. Additionally, the reduction rates for maxillary cysts tend to be lower compared to those for cystic lesions in the mandible [22]. The Caldwell–Luc approach remains a widely employed technique for addressing odontogenic cysts in the maxillary sinus, exhibiting minimal postoperative complications when performed judiciously [23]. The operation facilitates the prompt and successful extraction of pathological alterations situated within the maxillary sinus, and it is typically accompanied by mild and transient complications, including facial swelling or paresthesia [24]. The duration of decompression for cysts of considerable size, however, may span months to years [25]. Given the urgency for the rapid resolution of ocular symptoms in our patient, compounded by a history of previous sinus surgery altering anatomical considerations, enucleation via the Caldwell–Luc approach emerged as the preferred surgical modality to ensure an expeditious resolution and avert potential enduring ocular sequelae.

In conclusion, we can say that intensive follow-up after surgical procedures on the maxillary sinus and odontogenic cysts is necessary to avoid possible relapse even after a long period of time. Moreover, in rare cases of retinal artery and vein occlusions in younger patients with no risk factors, it is necessary to expand diagnostic procedures and check on the adjacent regions such as the maxillary.

## Data Availability

The original contributions presented in the study are included in the article, further inquiries can be directed to the corresponding author/s.
